# Microstructural Variation and a Physical Mechanism Model for a Ti-55511 Alloy during Double-Stage Hot Deformation with Stepped Strain Rates in the β Region

**DOI:** 10.3390/ma14216371

**Published:** 2021-10-25

**Authors:** Dao-Guang He, Gang Su, Yong-Cheng Lin, Yu-Qiang Jiang, Zhou Li, Zi-Jian Chen, Xin-Tao Yan, Yu-Chi Xia, Yang-Chen Xie

**Affiliations:** 1 School of Mechanical and Electrical Engineering, Central South University, Changsha 410083, China; 193812038@csu.edu.cn (G.S.); gugeyouling@163.com (Y.-Q.J.); lizhou_industry@hotmail.com (Z.L.); zjchen@csu.edu.cn (Z.-J.C.); yanxintao@csu.edu.cn (X.-T.Y.); xiayuchi@csu.edu.cn (Y.-C.X.); 193811005@csu.edu.cn (Y.-C.X.); 2 State Key Laboratory of High Performance Complex Manufacturing, Changsha 410083, China; 3 Light Alloy Research Institute of Central South University, Changsha 410083, China

**Keywords:** hot deformation, constitutive model, microstructural change, titanium alloy

## Abstract

The microstructural variation and high-temperature flow features of a Ti-55511 alloy in the β region are studied by utilizing double-stage compression with a stepped strain rate. The results demonstrate that the stresses in the latter stage of hot compression markedly reduce as the strain at the previous stage or the strain rate at the previous/latter stage drops. Moreover, the annihilation/interaction of substructures is promoted, and the distinct refinement of the dynamic recrystallization (DRX) in the β grain can be found. However, the coarsening of the β grain and the consumption of dislocation substructures are accelerated at high temperatures. Furthermore, the principal DRX nucleation mechanism of the Ti-55511 alloy during double-stage compression with a stepped strain rate in the β region is discontinuous DRX. Additionally, by using the microstructural variation characteristics related to the forming parameters, a physical mechanism equation is modeled to forecast the forming features, the DRX fraction, and the size of the β grain in the investigated alloy. The forecasted results are in accordance with the tested results, indicating that the established model can accurately forecast the microstructure variation and flow features of the studied alloy.

## 1. Introduction

Because of their noble mechanical and fatigue properties, near-β titanium alloys are promising structural alloys for preparing aerospace components [1,2,3,4]. An excellent microstructure is one of the essential factors required to achieve high-quality titanium structures [5,6]. Hot forming is widely recognized as a cardinal method for optimizing the microstructures of titanium alloys [7]. Usually, the variation in processing parameters can remarkably affect the change of flow characteristics, and also abruptly influence the microstructures in titanium alloys [8]. So, an in-depth recognition of the variation mechanisms of microstructures, in order to model the high-temperature forming features of titanium alloys, is significant.

Normally, the changes in microstructure, including dislocation, substructures, and α/β grains, are greatly sophisticated in hot forming, and apparently affect the properties of titanium alloy structures [9,10,11,12,13,14,15]. Recently, numerous studies have reported on the microstructural variation mechanisms of titanium alloys during hot deformation [16,17,18]. Zhao et al. [19] found that the initial substructures could promote subgrain nucleation and rotation, and apparently arouse the DRX nucleation in the hot tensile of a TA15 titanium alloy. Meanwhile, Foul et al. [20] and Wu et al. [21] reported that the phase transformation between the α phase and β phase strictly affects the high-temperature dynamic softening of titanium alloys. Kumar et al. [22] observed that the globularization of the α phase for a Ti-55511 alloy was affected by hot-tensile parameters and the initial addition of boron. Luo et al. [23] found that the spheroidization of the α phase can be primarily attributed to groove/boundary migration and sub-grain rotation. Li et al. [24] proposed a unified model to quantitatively characterize the globularization of the α phase in a TC6 alloy during high-temperature compression. Furthermore, Matsumoto et al. [25] reported that the development of continuous dynamic recrystallization (CDRX) could be accelerated by increasing the forming temperature. Similarly, reducing the strain rate could improve the DRX behavior of a TC18 titanium alloy [26]. Lin et al. [27] and Jiang et al. [28] revealed that the dynamic spheroidization of the α phase and the DRX of the β phase in a Ti-6Al-4V alloy during hot tensile forming were greatly influenced by the original microstructures. Abbasi et al. [29] found that the main DRX nucleation mechanism of a Ti-13V-11Cr-3Al alloy was changed from CDRX to DDRX as the temperature rose from 930 °C to 1030 °C. Additionally, based on the variation mechanisms of DRX with respect to forming parameters, Tan et al. [30] offered a physical-based equation to strictly forecast the DRX characteristics of a Ti-55511 alloy.

In the last few years, some models have been made to observe the hot-forming features of alloys [31,32,33,34,35]. Via the relation between flow characteristics and processing parameters, phenomenological constitutive equations have been proposed to model the high-temperature forming features of alloys. Typical phenomenological models, e.g., the Arrhenius equation [36,37], the Arrhenius equation optimized by a GA algorithm [38], a modified Khan–Huang–Liang equation [39], and the Hensel–Spittel (HS) equation [40], were developed to strictly describe the forming features of different titanium alloys. Meanwhile, because of the high learning adaptability of artificial intelligence, Peng et al. [41] and Mosleh et al. [42] developed high-accuracy artificial intelligence models to forecast the flow behavior of a Ti60 alloy and Ti-2.5Al-1.8Mn alloy, respectively. Correspondingly, several artificial intelligence algorithms have been proposed to precisely model the high-temperature flow features of other titanium alloys, such as a Ti-2Al-9.2Mo-2Fe beta alloy [43], a Ti40 alloy [44], and a Ti600 alloy [45]. However, the two aforementioned types of constitutive models have difficulty clarifying the microstructural change mechanisms of alloys during high-temperature forming. To develop constitutive models which consider the effects of metallurgical evolution mechanisms, several physical mechanism-based (PMB) equations were proposed to describe the high-temperature flow features of alloys [46,47]. Two representative PMB models, including the viscoplastic flow equation [48] and the internal-state-variable equation [49,50], were established and utilized to exactly predict the hot-forming features of some titanium alloys. Additionally, the dislocation density correlated model, which considered the variation in dislocation, affected by processing parameters and the interaction of microstructures, was another critical PMB constitutive model [51]. The dislocation density correlated model was developed to strictly forecast the high-temperature forming features of different titanium alloys, e.g., a Ti-55511 alloy [52,53], a two-phase TA15 alloy [54], a TiAl alloy [55], and a Ti5553 alloy [56].

Above all, the microstructural variation mechanisms and flow features of titanium alloys during high-temperature deformation with a constant strain rate have been researched in numerous reports. However, in practice, the strain rate during the high-temperature deformation of titanium alloys is often non-constant, and so the microstructural change mechanisms and forming features are different from those observed at a constant strain rate. Therefore, in this paper, the variation features of the microstructures and the flow behavior of a Ti-55511 alloy during double-stage hot compression with stepped strain rates are investigated. A physical mechanism model is proposed to calculate the flow features and microstructures of the Ti-55511 alloy, and its forecasting precision is verified.

## 2. Material and Experiments

**Figure 1:**
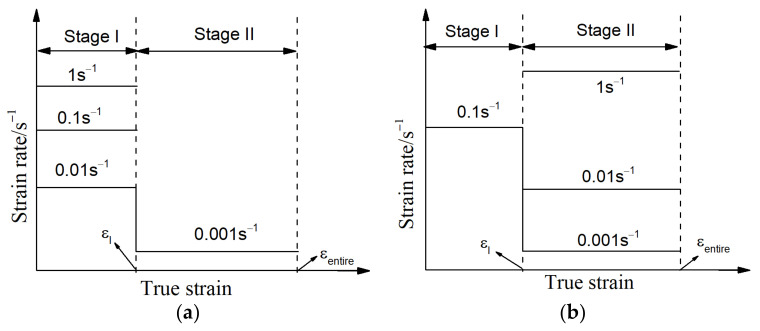
Experiment schemes of the researched alloy including: (**a**) type I: the strain rates changed from different values of ${\dot{\varepsilon }}_{I}$ into the constant ${\dot{\varepsilon }}_{\mathrm{II}}$, (**b**) type II: the strain rates changed from the constant ${\dot{\varepsilon }}_{I}$ into the different values of ${\dot{\varepsilon }}_{\mathrm{II}}$.

A commercial Ti-55511 titanium alloy with a chemical composition (wt.%) of 5.16Al-4.92Mo-4.96V-1.10Cr-0.98Fe-(bal.) Ti was used. The Ti-55511 titanium alloy was received as a wrought bar. Cylindrical specimens with a size of Φ8 mm × 12 mm (height) were made. The isothermal double-stage thermal compression tests were conducted using a Gleeble 3500 technique. Investigations into the microstructural changes and flow features of Ti-55511 titanium alloys during hot deformation with a constant strain rate are widely reported in previous works [47,52]. Nevertheless, the strain rate during the actual industrial manufacturing of parts is normally varied. Therefore, the experiment schemes used in order to research the high-temperature forming features of Ti-55511 titanium alloys at stepped strain rates in the β region are noted in Figure 1. Obviously, all tested cases consisted of two stages (I and II), and the strain rates ($\dot{\varepsilon }$) were different at stage I (${\dot{\varepsilon }}_{I}$) and stage II (${\dot{\varepsilon }}_{\mathrm{II}}$). Meanwhile, the deformation temperature (T) and entire strain ($\varepsilon_{\mathrm{entire}}$) were the constant values at each stage. The selected values of T were 890 °C, 920 °C, and 950 °C, respectively. Moreover, the value of $\varepsilon_{\mathrm{entire}}$ was 1.2. Because of the narrow high-temperature forming window, the strain rate of titanium alloys usually ranges from 0.001 s^−1^ to 1 s^−1^, as noted in previous studies [8,47,52]. So, in the present study, the selected values of ${\dot{\varepsilon }}_{I}$ and ${\dot{\varepsilon }}_{\mathrm{II}}$ were 0.001 s^−1^, 0.01 s^−1^, 0.1 s^−1^, and 1 s^−1^, respectively. The specific experimental process was performed as follows: the samples were firstly hot deformed at ${\dot{\varepsilon }}_{I}$ to the true strain of stage I ($\varepsilon_{I}$), and the strain rate was abruptly changed to ${\dot{\varepsilon }}_{\mathrm{II}}$. Then, the samples were further hot deformed to the true strain of $\varepsilon_{\mathrm{entire}}$. Here, the values of $\varepsilon_{I}$ were selected as 0.3, 0.6, and 0.9, respectively.

**Figure 2:**
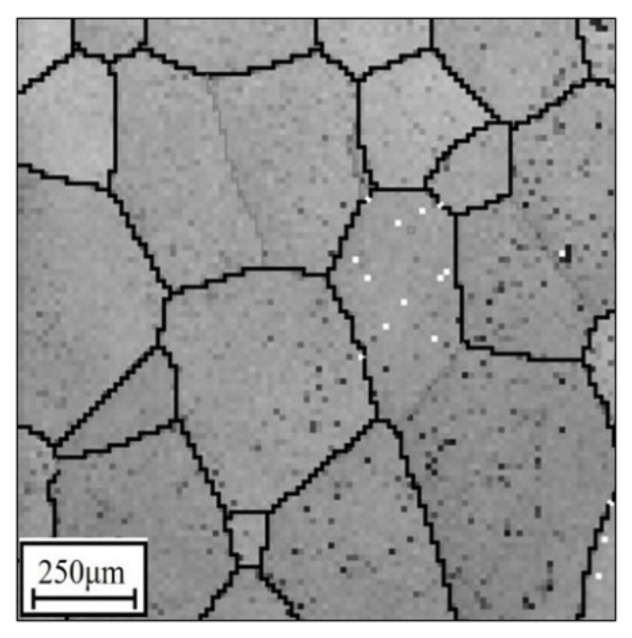
Original microstructures of the researched alloy (OIM image is indicated in a previous study [47]).

Prior to hot forming, the specimens were heated to the T at 10 °C/s, and kept there for 5 min. Once the hot compressive experiments were over, the specimens were promptly cooled in water. To understand the change mechanisms of the microstructures, techniques including electron backscatter diffraction (EBSD) were utilized. To analyze with EBSD, the hot-compressed specimens were machined in the direction of the hot deformation, and several thin sections were incised, ground, and polished to a thickness of 70 μm. Then, the thin sections were made into foils (Φ 3 mm) and etched using a solution (10 mL perchloric acid + 70 mL normal butanol + 120 mL methanol). The initial structure is displayed in Figure 2, and numerous equiaxed β grains can be observed.

## 3. Flow Characteristics and Microstructural Evolution during Double-Stage Hot Forming with Stepped Strain Rates

### 3.1. Flow Characteristics

**Figure 3:**
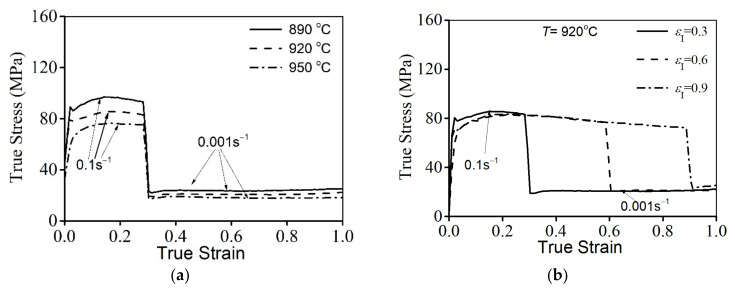

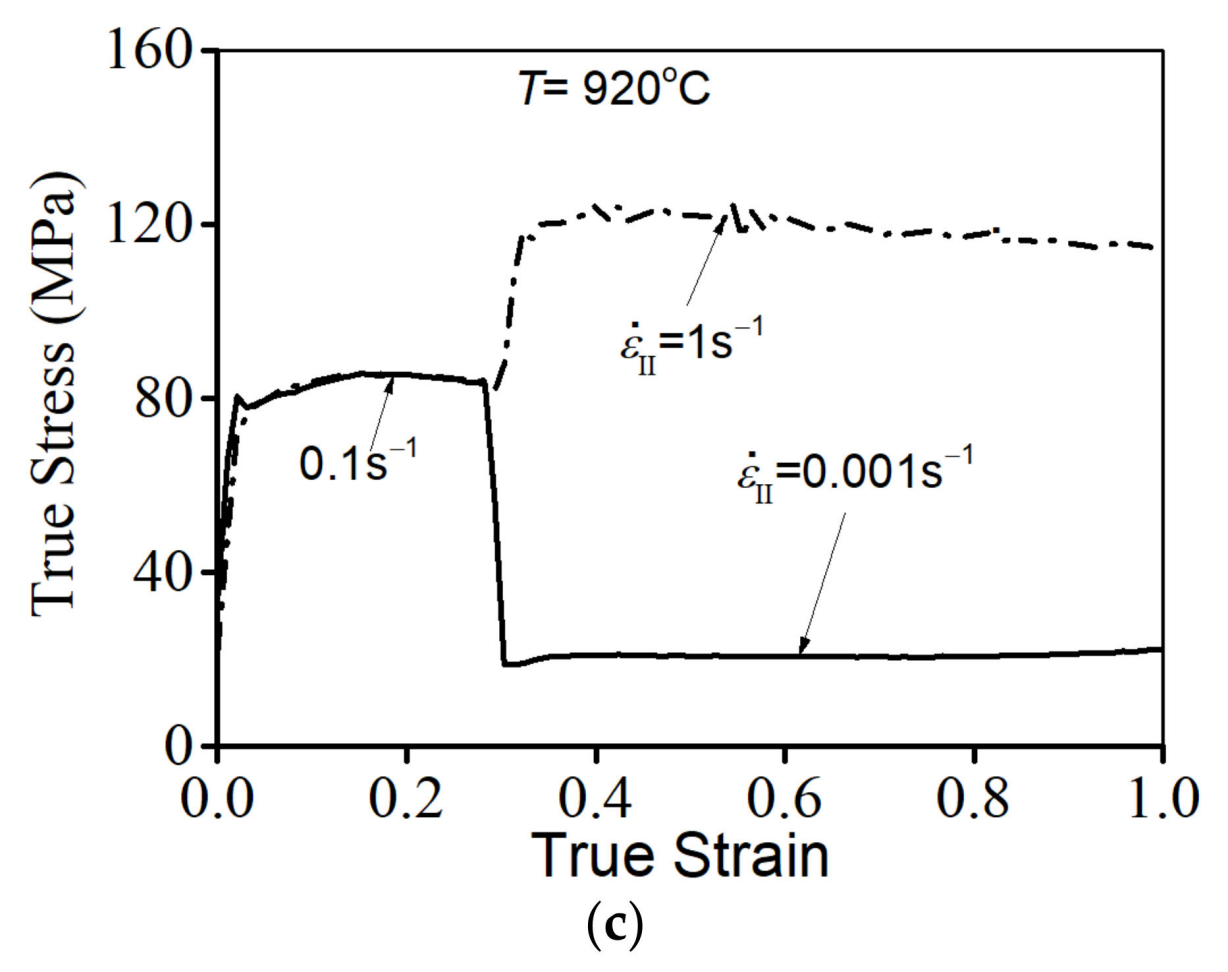
Flow characteristics at variations of: (**a**) T, (**b**) $\varepsilon_{I}$, (**c**) ${\dot{\varepsilon }}_{\mathrm{II}}$.

The variations in flow characteristics for the studied alloys during double-stage hot compression at stepped strain rates are depicted in Figure 3. Clearly, similar phenomena can be observed in all true stress–true strain curves. The true stress initially increases to a peak value as the true stain rises, and then progressively decreases. As the true stain exceeds $\varepsilon_{I}$, the strain rate changes from ${\dot{\varepsilon }}_{I}$ to ${\dot{\varepsilon }}_{\mathrm{II}}$, and the true stress is suddenly changed. Moreover, the evolution of flow behavior is greatly affected by the T, ${\dot{\varepsilon }}_{I}$, ${\dot{\varepsilon }}_{\mathrm{II}}$, and $\varepsilon_{I}$. As the T is reduced, the true stress abruptly increases, as noted in Figure 3a. Commonly, the nucleation/motion (slip and climb) of the dislocations/vacancies and the mobility of the grain boundary are accelerated at a high T. This can intensify the dynamic recovery and the nucleation/growth of dynamic recrystallization (DRX) grains, which can make the reduction in true stress increasingly obvious. As the $\varepsilon_{I}$ is decreased (Figure 3b), the flow stress at stage II of hot forming is slightly reduced. At the given $\varepsilon_{\mathrm{entire}}$, the true strain at stage II of the hot deformation is relatively increased with the reduction in $\varepsilon_{I}$. Increasing the true strain at stage II of the hot deformation can prolong the forming time for the nucleation and DRX grains at this hot forming stage. So, the flow stress is relatively reduced. Additionally, the flow stress at stage II of hot forming rises as the ${\dot{\varepsilon }}_{\mathrm{II}}$ is increased, as illustrated in Figure 3c. This is ascribed to the annihilation of dislocations/vacancies and the development of the DRX process, which benefits the dynamic softening effect.

### 3.2. Evolution of Microstructures

#### 3.2.1. Evolution of Grain Structures

**Figure 4:**
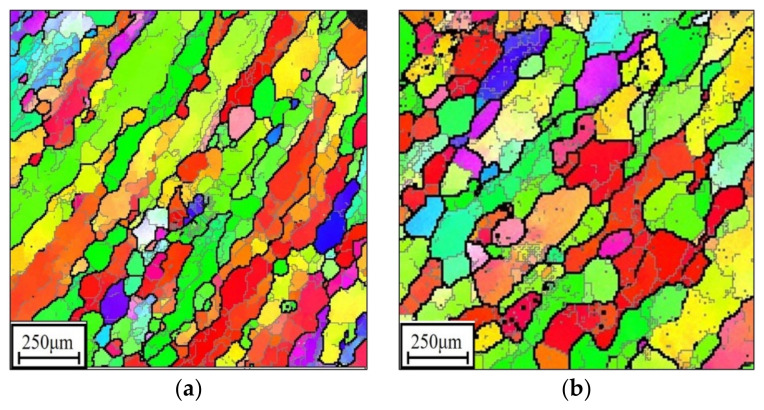

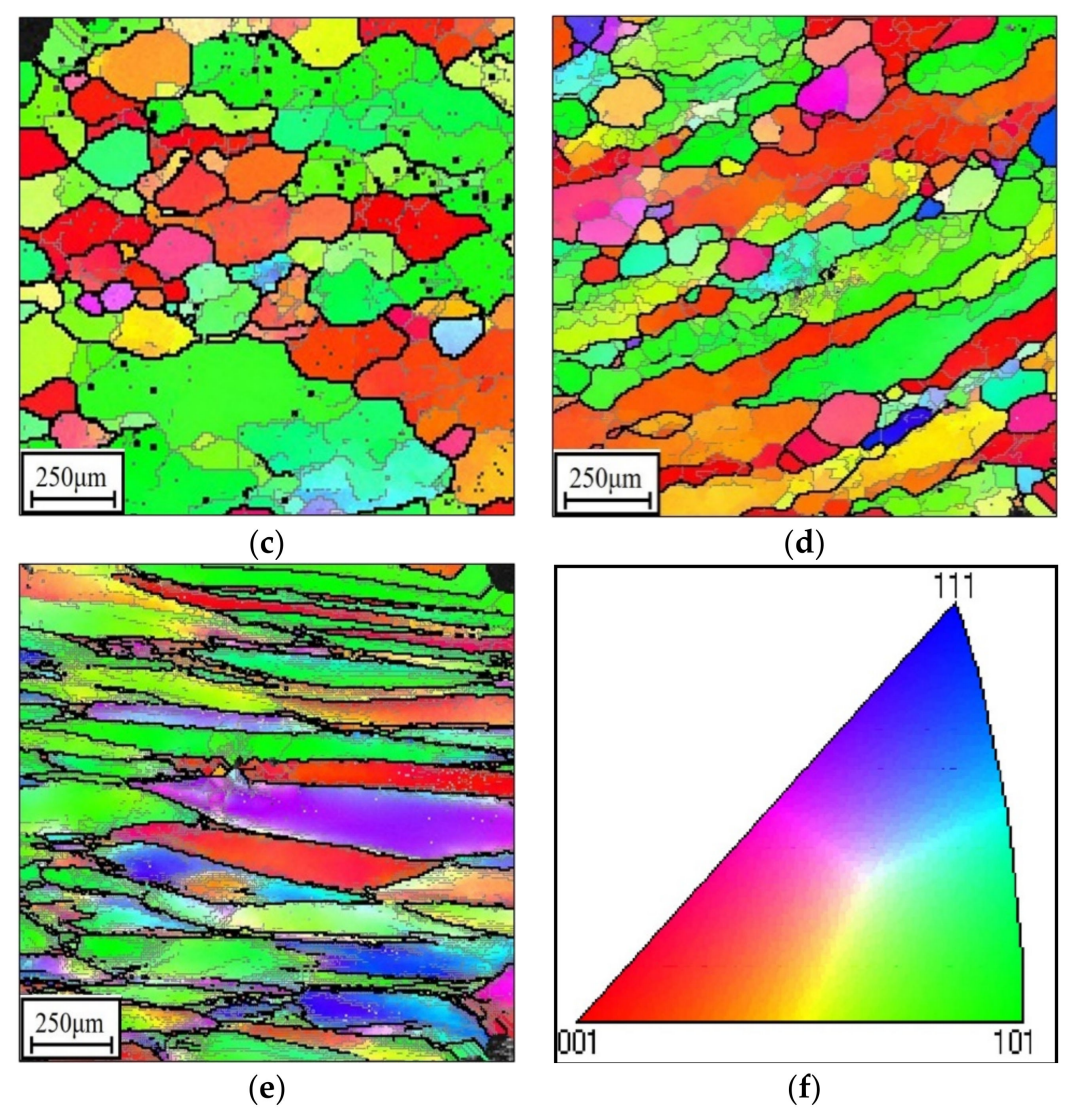
Orientation imaging microscopy maps of the investigated alloy at: (**a**) $890\;{}^{\circ}C/0.1\;s^{-1}/\varepsilon_{I}=0.3/0.001\;s^{-1}$, (**b**) $920\;{}^{\circ}C/0.1\;s^{-1}/\varepsilon_{I}=0.3/0.001\;s^{-1}$, (**c**) $950\;{}^{\circ}C/0.1\;s^{-1}/\varepsilon_{I}=0.3/0.001\;s^{-1}$, (**d**) $920\;{}^{\circ}C/0.1\;s^{-1}/\varepsilon_{I}=0.9/0.001\;s^{-1}$, (**e**) $920\;{}^{\circ}C/0.1\;s^{-1}/\varepsilon_{I}=0.3/1\;s^{-1}$, (**f**) IPF triangle image.

Changes in orientation imaging microscopy (OIM) maps at different double-stage experimental conditions are displayed in Figure 4. Clearly, the changes in grain structure are sensitive to the T, $\varepsilon_{I}$, ${\dot{\varepsilon }}_{I}$, $\varepsilon_{\mathrm{II}}$, and ${\dot{\varepsilon }}_{\mathrm{II}}$. For the researched alloy at the consistent experimental conditions of $\varepsilon_{I}$, ${\dot{\varepsilon }}_{I}$, $\varepsilon_{\mathrm{II}}$, and ${\dot{\varepsilon }}_{\mathrm{II}}$, the coarsening of DRX grains becomes obvious with increasing deformation temperatures, as indicated in Figure 4a–c. This is because the migration of vacancies/atoms is intensified at a high T, which induces an increase in the mobility of the grain boundary. Meanwhile, the DRX grains become more and more refined when the $\varepsilon_{I}$ increases (Figure 4b,d). This results from the number of substructures/DRX nucleation, substantially increasing with the increase in $\varepsilon_{I}$ during the first-stage hot deformation, thus causing the DRX grains to become refined during the second-stage hot deformation. Additionally, as the ${\dot{\varepsilon }}_{\mathrm{II}}$ increases to 1 s^−1^ (Figure 4e), the degree of DRX is markedly diminished, and the amount of residual original grains is increased. This is because the incubation time for the development of DRX is drastically reduced with the increase in ${\dot{\varepsilon }}_{\mathrm{II}}$, thus hindering the nucleation/coarsening of DRX grains.

#### 3.2.2. Evolution of Substructures

**Figure 5:**
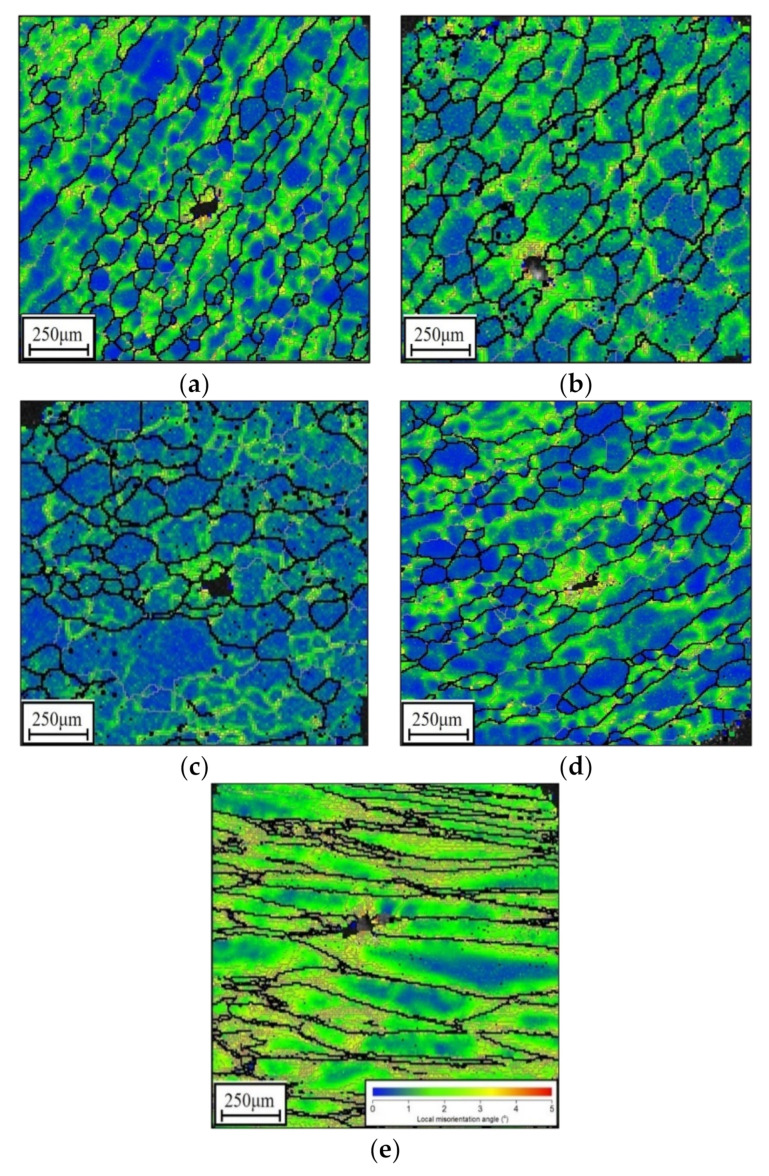
EBSD KAM maps at: (**a**) $890\;{}^{\circ}C/0.1\;s^{-1}/\varepsilon_{I}=0.3/0.001\;s^{-1}$, (**b**) $920\;{}^{\circ}C/0.1\;s^{-1}/\varepsilon_{I}=0.3/0.001\;s^{-1}$, (**c**) $950\;{}^{\circ}C/0.1\;s^{-1}/\varepsilon_{I}=0.3/0.001\;s^{-1}$, (**d**) $920\;{}^{\circ}C/0.1\;s^{-1}/\varepsilon_{I}=0.9/0.001\;s^{-1}$, (**e**) $920\;{}^{\circ}C/0.1\;s^{-1}/\varepsilon_{I}=0.3/1\;s^{-1}$.

Kernel average misorientation (KAM) images for the researched alloys at different tested conditions are shown in Figure 5. Here, the variations in KAM values are identified as changes in color. From Figure 5a–c, it may be noticed that the regions marked with yellow/red become narrowed with ascending *T*. The mean values of the KAM angle (${\bar{\theta }}_{\mathrm{KAM}}$) at 890 °C, 920 °C, and 950 °C are 0.966°, 0.792°, and 0.680°, respectively. This may be ascribed to the fact that increasing the *T* can drive preferential movement (cross-slip/climbing) and the annihilation of dislocations, along with the growth of subgrains, leading to the drop in substructures. Simultaneously, the shrinking of the area colored in blue (grain interior/boundaries) can be seen at the large value of $\varepsilon_{I}$, as displayed in Figure 5b,d. Accordingly, the value of ${\bar{\theta }}_{\mathrm{KAM}}$ is amplified from 0.792° to 0.851° as the value of $\varepsilon_{I}$ is increased from 0.3 to 0.9. This signifies that the increase in $\varepsilon_{I}$ can boost the nucleation/interaction of substructures, by means of restraining the annihilation of dislocations/vacancies and the propagation of the DRX grain boundary. Furthermore, as the ${\dot{\varepsilon }}_{\mathrm{II}}$ is increased, the expansion of regions colored in yellow/red becomes more and more obvious (Figure 5b,e), and the value of ${\bar{\theta }}_{\mathrm{KAM}}$ is raised to 1.15° (${\dot{\varepsilon }}_{\mathrm{II}}{=1\;s}^{-1}$). This results from the fact that increasing the ${\dot{\varepsilon }}_{\mathrm{II}}$ can shorten the hot forming time for the interaction and motion of dislocations/vacancies and inhibit the growth of substructures (dislocation cells/networks and subgrains).

#### 3.2.3. Evolution of Misorientation Angles and the DRX Nucleation Mechanism

**Figure 6:**
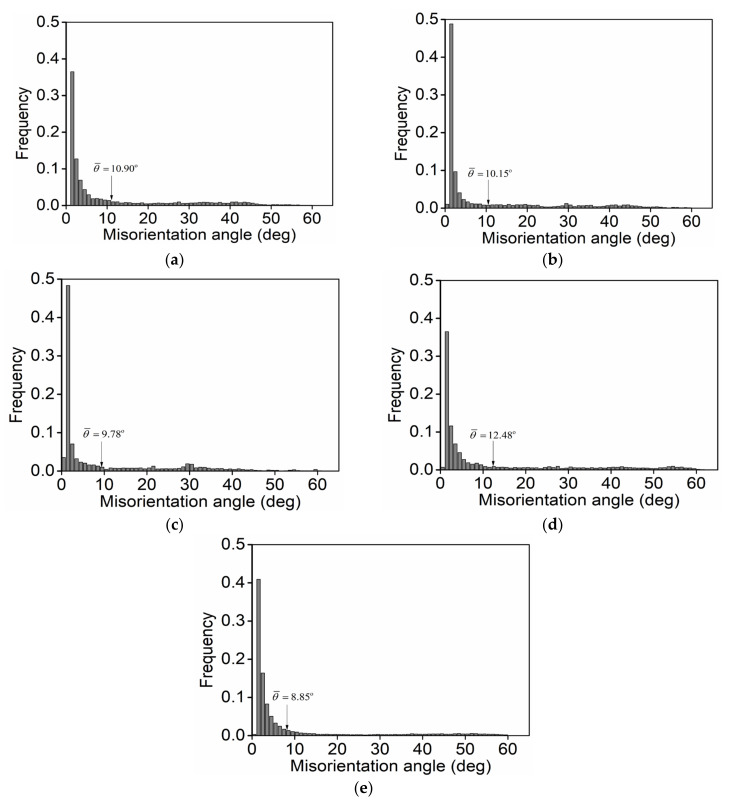
Misorientation angle scatters of the investigated titanium alloy at: (**a**) $890\;{}^{\circ}C/0.1\;s^{-1}/\varepsilon_{I}=0.3/0.001\;s^{-1}$, (**b**) $920\;{}^{\circ}C/0.1\;s^{-1}/\varepsilon_{I}=0.3/0.001\;s^{-1}$, (**c**) $950\;{}^{\circ}C/0.1\;s^{-1}/\varepsilon_{I}=0.3/0.001\;s^{-1}$, (**d**) $920\;{}^{\circ}C/0.1\;s^{-1}/\varepsilon_{I}=0.9/0.001\;s^{-1}$. (**e**) $920\;{}^{\circ}C/0.1\;s^{-1}/\varepsilon_{I}=0.3/1\;s^{-1}$.

For the investigated titanium alloy during double-stage thermal forming with stepped strain rates, the influence of the forming parameters on the mean misorientation angles of grain boundaries ($\bar{\theta }$) is demonstrated in Figure 6. It is noteworthy to mention that the value of $\bar{\theta }$ is reduced from 10.90° to 9.78° when the *T* is increased from 890 °C to 950 °C (Figure 6a–c). As described in Section 3.2.1, a high *T* can spur the growth of DRX nucleation. Normally, the coarsening of DRX grains is always accompanied by the consumption of refined grains via the expansion of the DRX grain boundary. This leads to a reduction in $\bar{\theta }$. Moreover, the value of $\bar{\theta }$ is increased with the increase in $\varepsilon_{I}$, as illustrated in Figure 6b,d. This is largely due to the number of high-angle grain boundaries (HAGBs) abruptly raised at high $\varepsilon_{I}$. Additionally, as the ${\dot{\varepsilon }}_{\mathrm{II}}$ is amplified from 0.001 s^−1^ to 1 s^−1^, the value of $\bar{\theta }$ is decreased from 10.15° to 8.85°, as noted in Figure 6b,e. This result is related to the fact that the nucleation/coarsening of DRX grains is restrained (Figure 4) and the nucleation of substructures is aggravated (Figure 5) when the ${\dot{\varepsilon }}_{\mathrm{II}}$ is raised.

**Figure 7:**
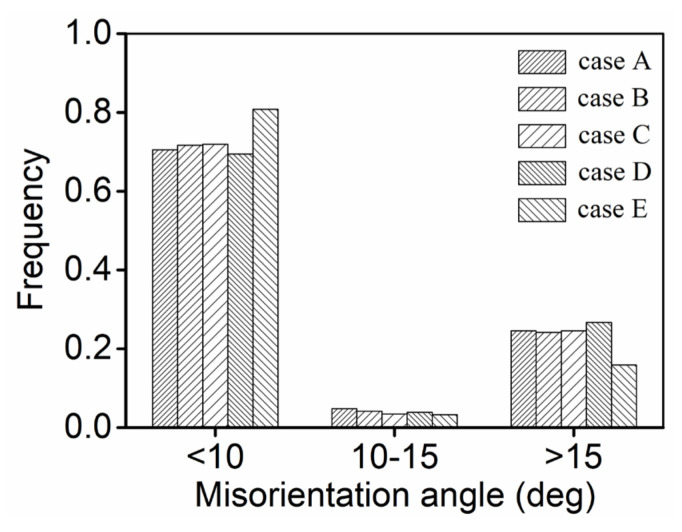
Frequency of misorientation angles during double-stage hot forming. (Here, cases A, B, C, D, and E are represented as the tested conditions of $890\;{}^{\circ}C/0.1\;s^{-1}/\varepsilon_{I}=0.3/0.001\;s^{-1}$, $920\;{}^{\circ}C/0.1\;s^{-1}/\varepsilon_{I}=0.3/0.001\;s^{-1}$, $950\;{}^{\circ}C/0.1\;s^{-1}/\varepsilon_{I}=0.3/0.001\;s^{-1}$, $920\;{}^{\circ}C/0.1\;s^{-1}/\varepsilon_{I}=0.9/0.001\;s^{-1}$. $920\;{}^{\circ}C/0.1\;s^{-1}/\varepsilon_{I}=0.3/1\;s^{-1}$, respectively.).

Commonly, the main DRX nucleation mechanisms for titanium alloys in hot forming are categorized as CDRX and discontinuous DRX (DDRX) [53,57]. In hot forming, the microstructural features of the titanium alloy changed by CDRX and DDRX are described as the serration/bowing of the grain boundary and the rotation of subgrains, respectively. According to Figure 4 and Figure 5, not only grain boundary bowing but also subgrain nucleation/rotation occurs, which suggests the appearance of two DRX nucleation mechanisms including CDRX and DDRX. To understand the principal nucleation mechanisms of DRX, the change in misorientation angle is statically analyzed, as shown in Figure 7. Distinctly, the difference in misorientation angle within the scope of 10–15° at all experimental conditions is small. So, it can be concluded that the principal nucleation mechanism of the DRX of the investigated alloy during double-stage thermal forming with stepped strain rates is DDRX.

## 4. The Physical Mechanism Constitutive Model

As indicated in Section 3, the changes in the microstructures, including dislocation, substructures, and grain, can be observed. The evolution and interaction of microstructures can induce the activation of intricate metallurgical mechanisms, i.e., work-hardening (WH), DRV, and DRX, which then leads to the changes in flow behaviors. Therefore, the change in flow stress for the alloy during hot deformation can be given as [47,52]:

(1) $$\sigma =\sigma_{y}+\sigma_{\rho }$$

where σ is flow stress, $\sigma_{y}$ is a short-range component, and $\sigma_{\rho }$ is dislocation interaction stress.

### 4.1. Identification of $\sigma_{y}$

Generally, the variations in forming parameters (T and $\dot{\varepsilon }$) can greatly affect the mobility and interaction of dislocation during hot deformation, and thus lead to a significant change in $\sigma_{y}$. $\sigma_{y}$ can be expressed as [52]:

(2) $$\sigma_{y}=A_{y}{\left(\dot{\varepsilon }\exp(\frac{Q_{y}}{RT})\right)}^{B_{y}}$$

where, $A_{y}$, $B_{y}$, and $Q_{y}$ are the material constants and R notes a gas constant.

The material constants in Equation (2) can be determined based on the computation methods reported in previous studies [52]. By the experimental data, the value of $A_{y}$, $B_{y}$, and $Q_{y}$ can be evaluated by a similar method related to previous studies [57,58,59]. $\sigma_{y}$ is given as:

(3) $$\sigma_{y}=1.589{\left(\dot{\varepsilon }\exp(\frac{205800}{RT})\right)}^{0.2052}$$

**Figure 8:**
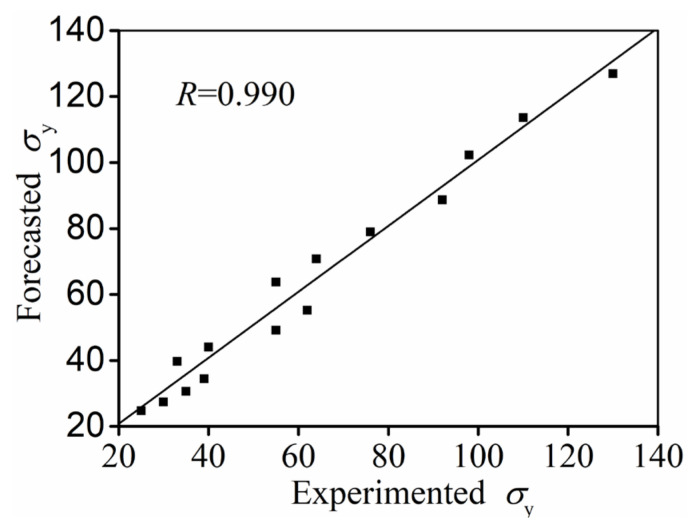
Correlation analysis between forecasted and experimented values of $\sigma_{y}$.

The relation coefficient (R) between the forecasted $\sigma_{y}$ and the experimented value is 0.990 (Figure 8). Therefore, the value of $\sigma_{y}$ for the investigated alloys can be forecasted by Equation (3).

### 4.2. Identification of $\sigma_{\rho }$

Commonly, the variation in dislocation for alloys during thermal plastic deforming is great and abruptly affects the $\sigma_{\rho }$. The value of $\sigma_{\rho }$ can normally be given as [52]:

(4) $$\sigma_{\rho }=M\alpha \mu b\sqrt{\rho_{i}}$$

where M notes the Taylor factor (3.06), α illustrates the constant (0.5), b indicates a burger vector with a value of 2.86 × 10^−10^ m^−1^, and μ expresses a shear modulus correlated with *T*. For the investigated titanium alloy hot compressed in the β region, μ is formulated as μ=21.8847−0.0153T [47]. $\rho_{i}$ is the dislocation density.

As the stress surpasses $\sigma_{y}$, the slip systems of dislocation are abruptly activated. The dislocation emergence and diminishment are intensified with gradual strain, and then affect the evolutional rate of the dislocation density. The depletion of dislocation is mainly caused by DRV and DRX during hot compression in the β region. So, $\rho_{i}$ can be given as:

(5) $${\dot{\rho }}_{i}={\dot{\rho }}_{i}{}^{+}-{\dot{\rho }}_{i}{}^{\mathrm{DRV}}-{\dot{\rho }}_{i}{}^{\mathrm{DRX}}$$

where ${\dot{\rho }}_{i}{}^{+}$ indicates the dislocation density emergence rate with respect to WH, and ${\dot{\rho }}_{i}{}^{\mathrm{DRV}}$ and ${\dot{\rho }}_{i}{}^{\mathrm{DRX}}$ are the dislocation density variation rate related to DRV and DRX, respectively.

The proliferative and cumulative rate of dislocation density is usually expressed as:

(6) $${\dot{\rho }}_{i}{}^{+}=\frac{1}{b\Lambda }\dot{\varepsilon }$$

where Λ illustrates the mean-free path of dislocation.

During hot compression in the β region, the value of Λ is primarily associated with average grain size ($d_{i}$) and substructure size (s). So, Λ can be described as:

(7) $$\frac{1}{\Lambda }=\frac{1}{s}+\frac{1}{d_{i}}$$

According to the evolution mechanism of substructures, s can be expressed as [52,53]:

(8) $$s=\frac{F_{s}}{\sqrt{\rho_{i}}}$$

where $F_{s}$ indicates the constant correlated with $\dot{\varepsilon }$ and T.

Then, $F_{s}$ is given as:

(9) $$F_{s}=A_{s}{\left(\dot{\varepsilon }\exp(\frac{-Q_{s}}{RT})\right)}^{B_{s}}$$

where $A_{s}$, $B_{s}$, and $Q_{s}$ are the material constants.

Generally, for hot forming at a high T, two metallurgy factors including grain coarsening and grain refinement by DRX are mainly attributed to a change in average grain size (d). The coarsening rate of grains during hot deformation can be modeled as [60]:

(10) $${\dot{d}}_{g}=A_{g}d^{-B_{g}}$$

where $A_{g}$ and $B_{g}$ are the material constants.

Meanwhile, the changed rate of grain size induced by DRX is represented as [60]:

(11) $${\dot{d}}_{x}=-A_{d}d^{B_{d}}{\dot{X}}^{C_{d}}$$

where $A_{d}$, $B_{d}$, and $C_{d}$ are the material constants.

By Equations (10) and (11), the change rate of the grain size can be summarized as:

(12) $${\dot{d}}_{i}={\dot{d}}_{x}+{\dot{d}}_{g}$$

Dislocation reset and disappearance induced by a DRV mechanism are strongly associated with the forming parameters. Therefore, ${\dot{\rho }}_{i}{}^{\mathrm{DRV}}$ can be represented as:

(13) $${\dot{\rho }}_{i}{}^{\mathrm{DRV}}=A_{v}{\left(\dot{\varepsilon }\exp(\frac{Q_{v}}{RT})\right)}^{B_{v}}\rho$$

where $A_{v}$, $B_{v}$, and $Q_{v}$ are the material constants.

Moreover, the DRX grain nucleation and growth are the two primary metallurgical mechanisms in the DRX process, and both are involved with the emergence, consumption, and interaction of dislocations. Correspondingly, the dislocation density change rate induced by DRX can be represented as:

(14) $${\dot{\rho }}_{i}{}^{\mathrm{DRX}}=\frac{A_{x}{\left(\dot{\varepsilon }\exp(\frac{-Q_{x}}{RT})\right)}^{B_{x}}\dot{X}\rho_{i}}{{\left(1-X\right)}^{C_{x}}}$$

where $A_{x}$, $B_{x}$, $Q_{x}$, and $C_{x}$ are material constants, and X represents the DRX fraction. The changed rate of the DRX fraction ($\dot{X}$) can be characterized as [52]:

(15) $$\dot{X}=\frac{A_{x2}M_{b}P{\left[X(1-X)\right]}^{B_{x2}}{\dot{\varepsilon }}^{C_{x2}}}{d^{D_{x2}}}$$

where $A_{x2}$, $B_{x2}$, $C_{x2}$, and $D_{x2}$ are the material constants. The grain boundary movement rate ($M_{b}$) and driving force (P) are usually characterized as [52]:

(16) $$M_{b}=\frac{D_{\mathrm{ob}}\delta b}{kT}{\left[\dot{\varepsilon }\exp(\frac{-Q_{\mathrm{Mb}}}{RT})\right]}^{B_{b}}$$

(17) $$P=\frac{\rho_{i}\mu b^{2}}{2}$$

where $Q_{\mathrm{Mb}}$ and $B_{b}$ are material constants, $D_{\mathrm{ob}}$ indicates the factor of self-diffusion, and δ represents the grain boundary thickness. $D_{\mathrm{ob}}\delta$ can be evaluated as 5.4 × 10^−17^ $m^{3}/s$ [52].

### 4.3. Verification of Physical Mechanism Constitutive Model

Several material constants should be firstly determined to develop a constitutive model with high forecasting precision, as noted in Equations (1)–(17). To identify the optimum material constants in the current context, a genetic algorithm is utilized. The optimized results of the constitutive model constants are illustrated in Table 1.

**Figure 9:**
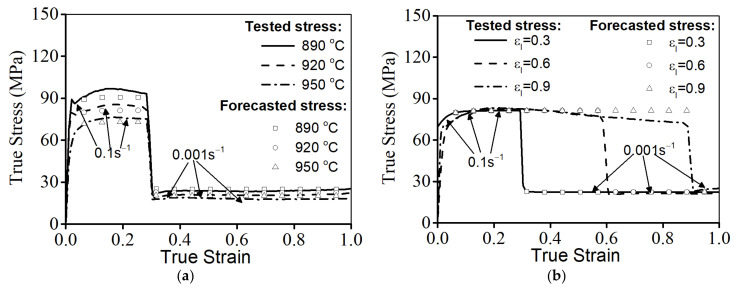

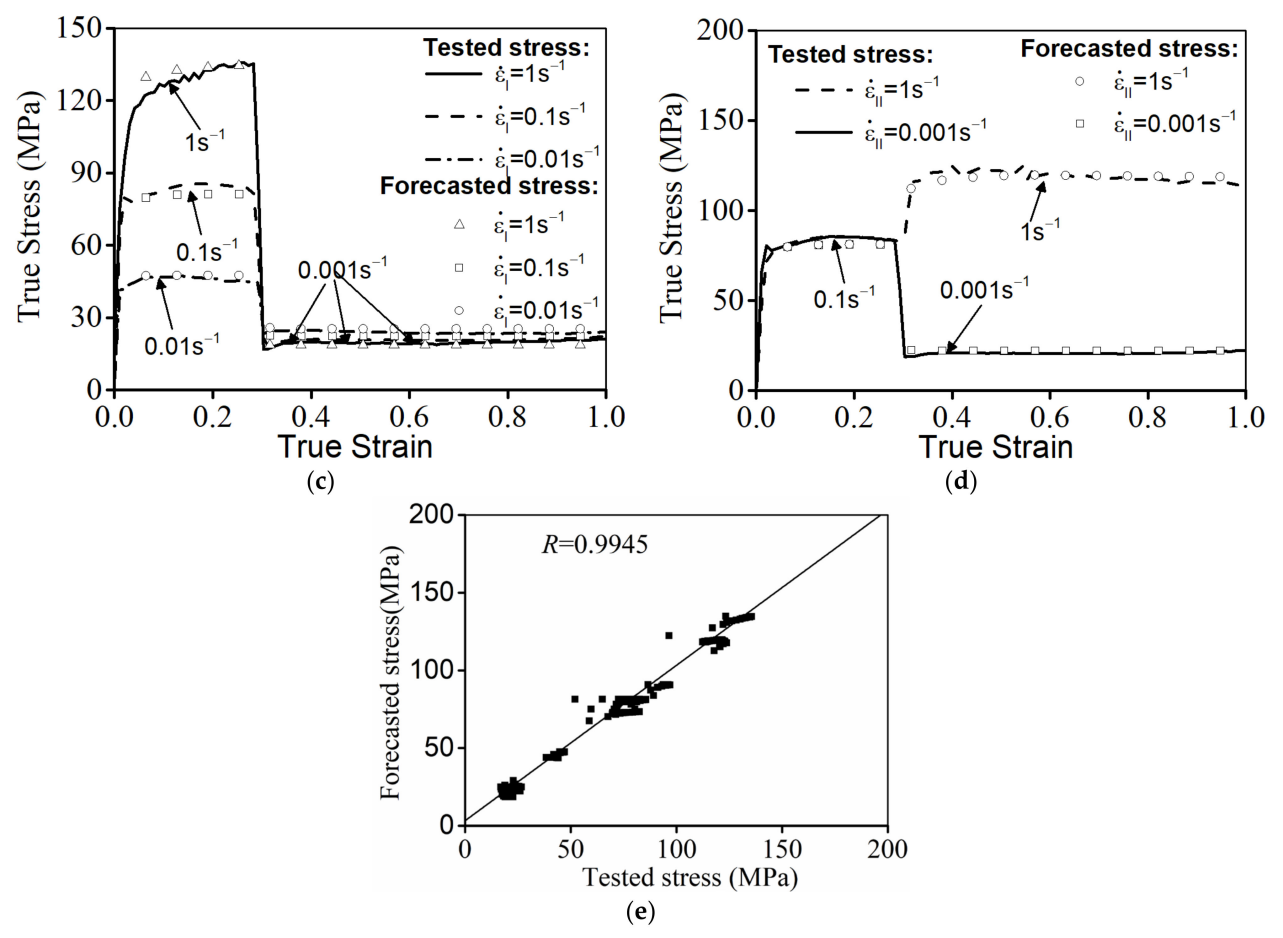
Comparison between forecasted and tested stress at: (**a**) T, (**b**) $\varepsilon_{I}$, (**c**) ${\dot{\varepsilon }}_{I}$, (**d**) ${\dot{\varepsilon }}_{\mathrm{II}}$, (**e**) relevant coefficient.

**Figure 10:**
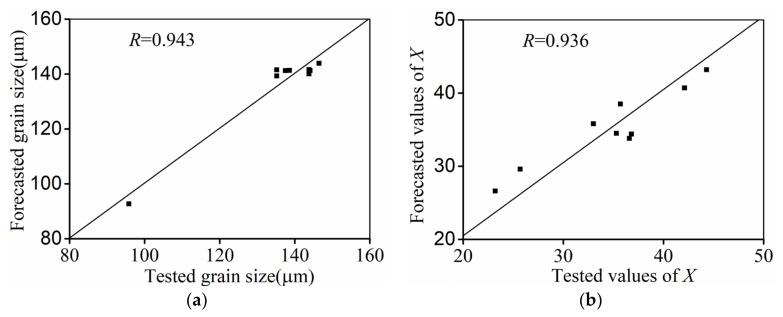
Comparison between forecasted and tested values of: (**a**) grain size, (**b**) *X*.

Comparisons of the forecasted values (σ, d, and X) with those of the test results are illustrated in Figure 9 and Figure 10. Distinctly, the influence of metallurgical mechanisms including WH, DRV, and DRX on flow behaviors in all hot-forming conditions can be seen, as noted in Figure 9. Moreover, it can be observed that the predicted values of σ in most experimental conditions are well matched to the tested values of σ, though a minor difference between the forecasted σ and the tested values can be observed at a $\varepsilon_{I}$ of 0.9. This certifies that the developed constitutive equations can forecast the flow features of a Ti-55511 alloy over a wide range. By the experimental data, the relevant coefficient (R) is identified as 0.9945 (Figure 9), which is in agreement with that of the developed models in previous studies [47,60]. This proves that the developed model can forecast the hot-forming features of the researched alloy. Additionally, it can also be observed that the forecasted values of d and X accord well with the experimental values (Figure 10), illustrating the high forecasting precision for the microstructural changes of the investigated alloy.

## 5. Conclusions

The changes in the microstructural and flow features of a Ti-55511 alloy during double-stage thermal compression with stepped strain rates were investigated. A physical mechanism constitutive model was established. Several principal findings can be summarized as:

1. The change mechanism of microstructures is closely influenced by the forming parameters. For a Ti-55511 alloy hot compressed at stepped strain rates, the DRX fraction was substantially increased with an increase in *T.* However, the DRX fraction noticeably dropped with increasing strain at stage Ι or strain rate at stage Ι/ΙΙ.
2. With increases in temperature, the coarsening of subgrains/β grains was simultaneously promoted. However, the mean size of subgrains/β grains was distinctly decreased at a large strain rate at stage Ι/ΙΙ or strain at stage Ι, because the boundary migration of subgrains/β grains was restricted at a short forming duration.
3. According to the microstructural changes related to the forming parameters, a physical mechanism-based constitutive model was developed. The relevant coefficients of the predicted stress, grain size, and the DRX fraction and those of the tested results were 0.9945, 0.943, and 0.936, respectively, suggesting the outstanding forecasting capability of the developed model.

## Figures and Tables

**Table 1:** The calibrated material parameters.

Material Parameter	Value	Material Parameter	Value
$A_{s}$	4.1797	$A_{x}$	0.7313
$B_{s}$	−0.2843	$B_{x}$	0.0137
$Q_{s}(\mathrm{kJ}/\mathrm{mol})$	6.4278	$C_{x}$	2.0210
$A_{g}$	2.3866	$Q_{x}(\mathrm{kJ}/\mathrm{mol})$	9.1687
$B_{g}$	−0.4468	$A_{x2}$	6.0877
$A_{d}$	0.7803	$B_{x2}$	−1.4294
$B_{d}$	0.0072	$C_{x2}$	−2.6513
$C_{d}$	0.9906	$D_{x2}$	0.5733
$A_{v}$	47.0321	$Q_{\mathrm{mb}}(\mathrm{kJ}/\mathrm{mol})$	0.1418
$B_{v}$	−0.1259	$B_{b}$	0.0045
$Q_{v}(\mathrm{kJ}/\mathrm{mol})$	0.04718		

## Data Availability

The raw/processed data required to reproduce these findings cannot be shared at this time as the data also forms part of an ongoing study.
